# Viruses in Extreme Marine Environments and Their Potential Existence in Extraterrestrial Environments

**DOI:** 10.3390/v18040457

**Published:** 2026-04-10

**Authors:** Andrew McMinn, Yantao Liang, Ziyue Wang, Min Wang

**Affiliations:** 1 Institute for Marine and Antarctic Studies, University of Tasmania, Hobart, TAS 7000, Australia; 2 College of Marine Life Sciences, Ocean University of China, Qingdao 266000, China; liangyantao@ouc.edu.cn (Y.L.); wzy6636@stu.ouc.edu.cn (Z.W.); mingwang@ouc.edu.cn (M.W.)

**Keywords:** virus, extraterrestrial, extremophile, sea ice, hydrothermal vent, trench

## Abstract

Viruses are abundant and widespread in extreme marine environments, such as sea ice, hydrothermal vents, and ocean trenches. They occur at temperatures up to 122 °C and down to −30 °C and pressures exceeding 100 MPa. Their distribution in these environments is closely correlated with that of their extremophile hosts, which are mostly bacteria, archaea, and microeukaryotes. Viruses have been shown to be capable of long-term survival in conditions simulating interstellar conditions. However, for them to reproduce, they would still need a host. Many recent astro-biological investigations have focused on habitability, specifically the ability of a planet to support the activity of at least one lifeform. The most likely candidates for extraterrestrial habitability in our solar system are the sea ice moons of Jupiter and Saturn, namely Europa and Enceladus. These are both thought to contain subsurface oceans of liquid water and potentially access to the necessary elements for microbial growth. If microorganisms were to be detected in these extraterrestrial environments, viruses might also be found coexisting with their host cells.

## 1. Introduction

**Figure 1:**
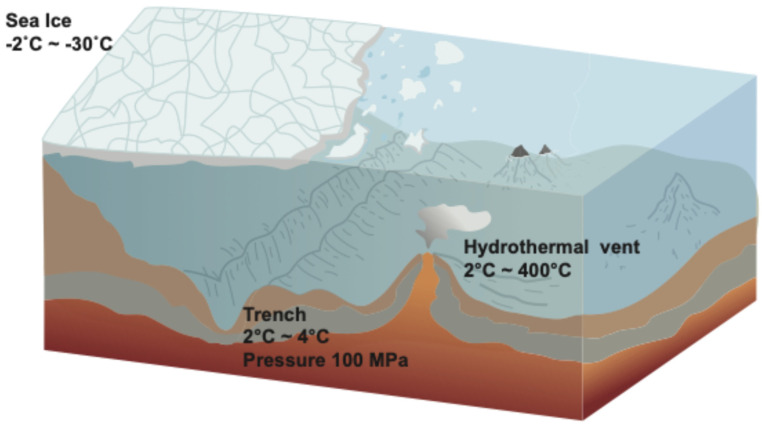
Extreme marine environments, sea ice, deep-sea trenches, and hydrothermal vents (original figure). Areas colored white indicate sea ice, blue represents sea water, light orange and grey represent bedrock and red rand dark orange represents magma.

Viruses are ubiquitous in all marine environments, with a global abundance thought to exceed 10^30^ [1]. They are not only abundant in the relatively benign ocean-surface environments but also in extreme environments such as deep-sea trenches, polar sea ice, and hydrothermal vents. Extremes of low and high temperatures are present in sea ice and hydrothermal vents, respectively, while extreme pressures are present in deep-sea trenches (Figure 1). Virus abundance and distribution are intricately entwined with those of their hosts, so it is unlikely that viruses will be present under physical conditions that are unsuitable for microbial life. So far, however, with the exception of ‘black smoker’ hydrothermal vents, where temperatures can exceed 400 °C, no marine environments have yet been identified where life is totally absent [2].

Bacteria and archaea have been found in natural environments with a temperature range of −30 °C to 122 °C [3,4], although the actual minimum temperature for microbial activity is ~−20 °C [2] and the maximum of 122 °C [5]. Likewise with pressure, bacteria and archaea have been found on the bottom of ocean trenches, at depths > 10,000 m, where pressures can exceed ~100 MPa, although under experimental conditions they have been found to survive many times this level [6]. Similarly, in experiments using simulated interstellar conditions, including high vacuum (i.e., very low pressure) and irradiation, many bacteria and viruses have been found to have high survival rates over extended periods of time [7]. Viruses associated with these extremophile microbes have been found in all of these extreme environments (Figure 1).

Viruses lack the ribosomal proteins necessary for self-replication and instead manipulate the host cell’s ribosomes for viral protein synthesis. However, recent studies have identified both prokaryotic [8] and eukaryotic [9] viruses that can encode ribosomal proteins. These discoveries emphasize the complexity and uncertainty regarding the origin and evolution of viruses. Currently, all known viruses parasitize cells, and so it is likely that cellular lifeforms would have needed to have evolved before viruses could have developed [10]. Thus, in the context of the existence of viruses elsewhere in the universe, it would be critical that viral host cells were also present. However, some have suggested that viruses or virus-like elements capable of replication may have emerged from abiotic lipid-coated micro-cavities of hydrothermal vents that allowed replication biochemistry and ultimately led to the emergence of a prokaryotic cell with viruses or virus-like elements [11]; however, the actual origin of viruses remains unknown.

Viruses come in four basic types: double- and single-stranded DNA viruses and double- and single-stranded RNA viruses. While DNA viruses have been studied for longer and are better known, research on RNA viruses is expanding rapidly [12]. Viruses mainly adopt one of two basic life strategies. In the ‘lytic’ strategy, viruses infect the host cell and subsequently cause cell death, releasing the virus particles into the environment. In the ‘lysogenic’ strategy, viruses infect the host but integrate their genome into that of their host. When the host reproduces and copies its genome, the integrated viral genome is also replicated. Lytic life strategies were previously thought to predominate in eutrophic environments, while lysogenic life strategies were more prevalent in oligotrophic environments. However, Knowles et al. [13] proposed that a piggyback-the-winner model, wherein lysogenic dynamics become increasingly important in ecosystems with high microbial densities, was also common. In this paper, we have used the traditional morphology-based taxonomy to be consistent with the cited references.

## 2. Review

### 2.1. Sea Ice Environments

Sea ice environments are mostly ephemeral, forming in autumn and melting in spring. While habitats at the underside of the ice, at the ice-water interface, are relatively benign with small temperature and salinity fluctuations, those at the surface or within the ice in brine channels can be exposed to extremes in both temperature and salinity. In winter, brine channel temperature can drop to approximately −30 °C, and salinity can exceed 250 ppt [14,15].

Sea ice supports rich and diverse microbial communities [14,15,16,17]. Diatoms typically dominate the microalgal component, although dinoflagellates and prymnesiophytes can also be abundant [18]. Primary production, particularly in spring and early summer, can be high and significantly contribute to the productivity of the broader ecosystem. Photosynthesis has been measured at temperatures down to approximately −10 °C, although many taxa can survive at much lower temperatures [19].

Thirteen major bacterial taxonomic divisions have been found in sea ice [16]. Members of the Gammaproteobacteria and Bacteriodetes are dominant, but Alphaproteobacteria and members of the phyla Actinobacteria and Verrucomicrobia are also abundant [16].

Polar DNA viruses, including those from sea ice, have recently been reviewed by Zhai et al. [20]. While a large proportion of sea ice viruses remains unclassified, metagenomic and metatranscriptomic analyses have found that dsDNA viruses (e.g., *Caudoviricetes*) were most abundant, with smaller proportions of ssDNA, RNA, and nucleocytoplasmic large DNA viruses (NCLDV). A significant proportion were novel and endemic viruses [17]. Some of these viruses targeted chemolithoautotrophs, including ammonium-oxidizing archaea (*Nitrosopumilus* spp.) and sulfur-oxidizing bacteria (*Thioglobus* spp.), underscoring the important role of viruses in under-ice biogeochemical cycling [20]. Lytic viral infections predominate in sea ice environments [21].

Currently there is little information on the abundance, taxonomy and distribution of RNA viruses in sea ice environments, although they are likely to be similar to other polar marine environments [20].

### 2.2. Hydrothermal Vents

Hydrothermal vents occur in areas of active volcanism, primarily associated with mid-ocean ridges, areas where geological plates are actively diverging. Water is heated by magma in the subsurface and released at the surface, often forming distinctive, tubular structures known as chimneys. These waters are typically characterized by high temperatures, CO_2,_ and mineral content but low pH and oxygen [22]. There are two distinctly different types of hydrothermal vents: the ‘black smokers’ that occur above magma chambers and can have water temperatures exceeding 400 °C, and those known as ‘lost city’ type, which occur off the ridge and have temperatures of 50–90 °C.

Hydrothermal vents support a surprisingly high biomass and highly biodiverse communities. The walls of the chimneys that characterize these ecosystems are often encrusted with dense communities of tube worms, scale worms, palm worms, sulfide worms, and limpets. Bacterial 16S ribosomal RNA gene sequences correspond to a diverse community that includes species of Methylobacter (or *Methylomonas*), species of Thiomicrospira, members of the Firmicutes phylum, and *Desulfotomaculum alkaliphilus* Pikuta et al. 2000 [23,24].

Only a small proportion (~30%) of DNA viruses present in hydrothermal vents can be classified. Most of these have been found to be double-stranded DNA viruses with tails within the *Myoviridae* family. Single-stranded DNA (ssDNA) comprised only ~36% of the total, mostly belonging to the Microviridae family. CRESS-DNA (circular re-encoding ssDNA) viruses are represented by the family Cycloviridae [24]. Viruses from these environments showed a high degree of endemism, often even to individual vents, but unsurprisingly showed close correlations in abundance with their hosts [24]. *Candidatus thermoplasmatota* Rinke et al. 2019 was the most commonly predicted archaeal host, while Gammaproteobacteria was the most commonly predicted bacterial host [25].

Over 140 auxiliary metabolic genes (AMGs) participating in 34 metabolic pathways have been identified in vent viral communities. Those from the less extreme plume environment included genes for genetic information processing, metabolism of carbohydrates and amino acids, and lipid biosynthesis. Those from the vent itself include sulfur oxidation and sulfate reduction pathways. Also abundant is the thiouridine synthase subunit E gene (*tusE*, a homolog of *dsrC*). This gene modifies tRNA thiol to help bacteria survive at high temperatures by improving the stability of the tRNA structure [26].

### 2.3. Trench Environments

Deep-sea trenches (hadal zone) are primarily located at convergent geological plate margins and are usually associated with active subduction zones. They are most prevalent in the Pacific Basin, although some are also found in the northeastern Indian Ocean, the South Atlantic Ocean, and the Caribbean Sea. The deepest of these trenches is the Mariana Trench with a maximum known depth of 10,984 m.

Although there is a general reduction in abundance and diversity of marine life with water depth in trenches, sampling has identified a diverse community of metazoan organisms, including fish, holothurians, polychaetas, mollusks, amphipods, and more [27]. Diverse microbial ecosystems have also been reported from Mariana Trench sediments [24,28]. However, as these microbial genomes were derived from either sediments or dispersed in the water column, it is not immediately clear whether they represent in situ production or organic material sedimented from higher levels. Glud et al. [29], however, used an autonomous micro-profiling system to demonstrate that in situ microbial activity in trenches was actually quite high and that organic matter degradation was probably the main source of metabolic energy. Although most genomic studies of hadal zones have reported a high level of unknown organisms, the main prokaryotic phyla present were reasonably similar to other marine environments, with dominant bacterial taxa including Pseudomonadota, Chloroflexota, and Marinisomatota, as well as dominant archaeal taxa including Nanoarchaeota, Thermoplasmatota, and Thermoproteota [30,31,32]. Functionally, hydrocarbon-degrading bacteria comprised >40% of the abundance of 16S RNA genes, and the Alteromonadales and ammonia-oxidizing Thaumarchaeota are the dominant prokaryotic microbes in the hadal zone [33,34,35]. Pico- and nano-phytoplankton communities, comprising Dinoflagellata, Chrysophyceae, Haptophyta, Chlorophyta, Prochloraceae, Pseudanabaenaceae, Synechococcaceae, and Eustigmatophyceae, have also been identified [36]; however, as most of these are phototrophic, they were almost certainly exported from the surface. Interestingly, although these bacteria and archaea are living in a high-pressure environment, the taxonomic composition of the communities is similar to that elsewhere in the ocean, and so far, specific adaptations to this high-pressure environment have not been identified.

Several studies have used genomics to investigate the abundance, diversity, and distribution of viruses in ocean trench environments [33,35,37]. In general, most DNA viral sequences could not be classified, but of those that could be, *Myoviridae*, *Siphoviridae*, and *Podoviridae* were the most abundant. The most abundant predicted hosts for these taxa were the bacteria Gammaproteobacteria (more specifically Alteromonadales and Oceanospirillales [35] and the archaea Thaumarchaeota [33]. AMGs were mainly related to carbon, nitrogen, sulfur, and arsenic metabolism [33]. Lysogenic viral production has been found to predominate in trench environments [33].

Our recent work on RNA viruses from the Mariana Trench identified the *Totiviridae* family, the simplest known dsRNA viruses, in the phylum Duplornaviricota (50.72%) as the dominant virus taxon. RNA viral Operational Taxonomic Units (vOTUs) within this family identified viruses infecting fungi, such as *Phakopsora* and *Saccharomyces cerevisiae* Meyen ex E.C. Hansen, as dominant. So far, RNA viruses from sediments have not been classified.

## 3. Discussion

It has now been well demonstrated that viruses are present in some of the most extreme marine environments on Earth. It has also been shown that viruses can withstand the extreme environmental conditions thought to be present elsewhere in the universe. However, there are important differences between the ability to survive, grow, and thrive and being able to evolve in situ. Viruses are dispersed throughout the oceans, but they are only able to replicate within a host cell. Therefore, there is always a close association between the abundance and distribution of the virus and the abundance and distribution of its hosts.

When considering the likelihood of viruses being present in extraterrestrial environments, it may be assumed that they evolved after prokaryotic lifeforms or that they coevolved. As such, the search for extraterrestrial viruses is also the search for extraterrestrial microbial life.

The search for extraterrestrial life is largely based on the search for ‘habitable’ planets. Habitability has recently been defined as having a set of environmental conditions suitable for supporting the activity of at least one microorganism [38]. Life on earth has three basic requirements: liquid water, the availability of basic elements such as carbon, hydrogen, oxygen, nitrogen, phosphorus, sulfur, and a number of trace elements, and a source of energy [39]. Much attention has been given to the search for water worlds, but a distinction needs to be made between those with liquid water at the surface and those with subsurface water. The visionary early astrobiologist Carl Sagan is well known for popularizing the maxim ‘follow the water’ when it comes to looking for life elsewhere in the universe. Planets with liquid water on the surface can potentially sustain photobiology in an oxygenated environment. Those with only subsurface liquid water would require an alternative energy source to sunlight. On Earth, many such alternative sources and metabolic pathways have been identified, including methanogenesis, sulfur reduction, nitrification, iron oxide reduction, etc. [40]. Similarly, many viruses from these environments have been found to possess AMGs that code for these functions, potentially allowing horizontal gene transfer and, thus, conferring alternative metabolic pathways to their host.

Life on Earth almost certainly began in an anaerobic marine environment. Phototrophic lifeforms did not evolve until at least a billion years later. Microorganisms need water, key elements, and an energy source to metabolize. These prerequisites exist adjacent to many hydrothermal vent systems. Martin et al. [41] discussed chemical reactions in hydrothermal vents and considered the serpentinization reaction in some vents, which produce abiotic CH_4_ and short hydrocarbons, to be a possible model for the origin of life. It was also theorized that life probably originally developed in these environments. It is unclear how common these systems are elsewhere in the universe, but the probability of their existence is high.

The most promising sites for extraterrestrial life, including viruses, in our own solar system are considered to be the ‘ocean moons’ of Jupiter and Saturn, namely Europa and Enceladus. While there is evidence of water ice on our Moon and on Mars [42], there is better evidence for subsurface liquid water being present on these moons [42]. Furthermore, in 2005, the Cassini spacecraft detected the presence of complex organic molecules, potentially substrates for microbial growth, in ice plumes originating from the subsurface ocean of Enceladus [43].

**Figure 2:**
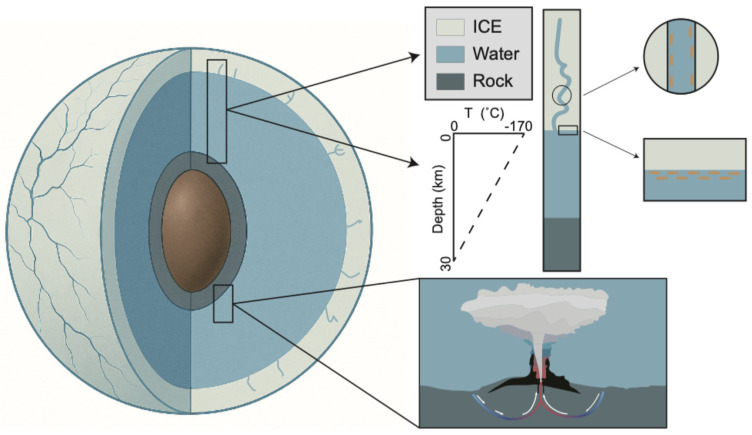
Potential microbial habitats on the icy moons of Jupiter and Saturn, i.e., Europa and Enceladus. Ice habitats include the ice-water interface and within channels. Hydrothermal habitats potentially exist at the base of the water column. Dashed line represents the temperature gradient between the surface and base of ocean. Figure modified from Martin and McMinn [40].

Martin and McMinn [40] suggested that there were two potential habitats for sea ice-like microbes in the oceans of Europa and Enceladus, one at the ice-water interface and another in fissures leading from the ocean to the surface (Figure 2). Modeling suggests that temperatures above −20 °C could extend up to 5 km from the ice-ocean interface through the 20–30 km thick ice crust [40]. Pressures at this depth are estimated to be between 84 and 205 MPa, i.e., within the range found in terrestrial trenches. However, even though there is sufficient light at the surface of the ice for photosynthesis, temperatures are below −170 °C. On Europa and Enceladus, water is thought to be maintained in the liquid state by geothermal heating resulting from tidal flexing caused by the eccentric orbits of these moons around their giant planets [44]. The presence of this geothermal energy might thus also imply the potential presence of hydrothermal plume-like environments (Figure 2).

It seems probable that Europa and Enceladus contain habitats that would allow the survival and perhaps even growth of microorganisms and viruses. It also seems likely that these environments have existed over geological time scales. These conditions thus potentially exist for the initiation and development of primitive life forms. Whether this has actually happened, though, will remain unknown until we look beneath the icy crusts of these moons. However, given the large number of likely habitable planets in the universe, the existence of life outside our solar system must also be reasonable.

## Data Availability

All data has been previously published.
